# Trends in HIV-related knowledge, behaviors and determinants of HIV testing among adolescent women aged 15–24 in Nigeria

**DOI:** 10.1186/s41182-025-00737-1

**Published:** 2025-06-11

**Authors:** Okoro Lenz Nwachinemere, Simon Nyegenye, Aaron Mwesigwa, Naya Gadzama Bulus, Jonathan Mawutor Gmanyami, Kaweesi Abdulrahim Mukisa, Isaac Isiko

**Affiliations:** 1 Department of Community Medicine, David Umahi Federal University Teaching Hospital, Uburu, Ebonyi Nigeria; 2 Department of Statistics and Applied Planning, School of Statistics and Planning, Makerere University, Kampala, Uganda; 3 Department of Pharmaceutical Sciences, Faculty of Health Sciences, Marwadi University, Rajkot, Gujarat 360003 India; 4 Department of Community Medicine, College of Medical Sciences, Abubakar Tafawa Balewa University, Bauchi, Bauchi Nigeria; 5 School of Medicine, College of Health Sciences, Makerere University, Kampala, Uganda; 6 Department of Community Medicine, Axel Pries Institute of Public Health and Biomedical Sciences, Nims University, Jaipur, Rajasthan India; 7 Global Health and Infectious Diseases Research Group, Kumasi Centre for Collaborative Research in Tropical Medicine, Kumasi, Ghana; 8 School of Public Health, Kwame Nkrumah University of Science and Technology, Kumasi, Ghana; 9 Department of Biomolecular Resources and Biolab Sciences, College of Veterinary Medicine Animal Resources and Biosecurity, Makerere University, Kampala, Uganda

**Keywords:** HIV, HIV knowledge, HIV testing, Young women, HIV trend analysis, HIV in Nigeria, MICS data

## Abstract

**Background:**

HIV remains one of the major global public health challenges, having claimed over 36 million lives so far, especially in sub-Saharan African countries like Nigeria. This study aimed to look into the trends in HIV-related knowledge, behavior and testing among young women in Nigeria.

**Methods:**

This study used data extracted from women aged 15–24 years who indicated that they had undergone HIV testing from the Nigeria Multiple Indicator Cluster Surveys (MICS) for 2007, 2011, and 2016. Across these surveys, similar sampling designs were applied, using a two-stage cluster sampling to generate a nationally representative sample of households. In the first stage, clusters were selected using the most recent available census from sampling frames. In the second stage, households were selected from each cluster. There was stratification of urban and rural for the different sampled clusters. The analysis was performed using STATA 17 software.

**Results:**

Northwest and South-South geopolitical zones, rural residential status and good knowledge about HIV were significantly associated with HIV testing. From 2011 to 2016, young women with primary education were significantly associated with reduced odds of HIV testing compared to those with at least secondary education. Young women with good behavior towards HIV prevention were significantly associated with higher odds of HIV testing, ranging from 1.7 times higher in 2011 to 1.8 times higher in 2016 compared with young women with poor behavior.

**Conclusion:**

By prioritizing education, integrating HIV education and testing into school-based programs, and increasing access to healthcare services in rural areas, we can empower adolescents to make informed decisions about their health and reduce the spread of HIV.

## Introduction

Young women in Nigeria continue to be disproportionately affected by the Human Immune deficiency Virus (HIV), with their vulnerability linked to multiple factors, including socioeconomic disadvantages, gender inequalities, and behavioral risks [1–3]. Despite extensive global efforts to curb new infections, young women aged 15–24 remain at a higher risk compared to their male counterparts. Studies show that women in this age group are three times more likely to acquire HIV than young men, a trend that mirrors findings from other sub-Saharan African countries [4, 5]. The burden of HIV among this population is exacerbated by factors such as low HIV-related knowledge, inconsistent condom use, multiple sexual partnerships, transactional sex, and limited access to testing and prevention services [6, 7]. HIV-related knowledge refers to understanding methods of HIV transmission, prevention strategies, and available treatments. HIV testing indicates whether young women have ever tested for HIV, critical for early diagnosis and treatment. HIV preventive behavior includes consistent condom use and limiting sexual partners. Despite Nigeria’s national HIV prevalence being approximately 1.4%, young women aged 15–24 experience higher infection rates, driven by limited knowledge, behavioral risks, and low testing uptake, underscoring the importance of targeted interventions.

Over the past two decades, significant investments have been made in HIV prevention and treatment programs in Nigeria [8–10]. However, trends indicate persistent knowledge gaps and a decline in comprehensive HIV awareness among young Nigerians, including women. Previous research suggests that comprehensive knowledge of HIV prevention decreased in key domains between 2003 and 2013, while risky sexual behaviors increased [11–13]. In particular, early sexual debut, unprotected sex, and low perception of HIV risk remain widespread among adolescent girls and young women [14, 15]. Although awareness of HIV prevention methods, including condom use and voluntary testing, has grown, stigma, discrimination, and limited access to healthcare services continue to hinder uptake [16–20].

HIV testing remains a critical component of prevention, yet testing rates among young women in Nigeria remain suboptimal. Studies show that a significant proportion of young women do not know their HIV status, despite being aware of the availability of free testing services. Barriers to testing include fear of stigma, lack of confidentiality, misconceptions about HIV transmission, and limited accessibility of voluntary counselling and testing (VCT) services [21, 22]. In many cases, adolescent girls and young women are also less likely to negotiate condom use, increasing their susceptibility to infection. Furthermore, disparities in HIV knowledge persist across different regions in Nigeria, with rural women and those from lower socioeconomic backgrounds demonstrating lower awareness levels compared to their urban counterparts [23–25].

The Prevention of Mother-to-Child Transmission (PMTCT) program remains a crucial intervention for reducing HIV transmission from infected mothers to their children. However, despite the increased availability of PMTCT services, acceptance remains low in Nigeria [26, 27]. Studies show that many women, particularly young mothers, do not access these services due to stigma, misinformation, and inadequate healthcare infrastructure [28, 29]. Without proper PMTCT interventions, HIV-positive women face a 35% chance of transmitting the virus to their infants during pregnancy, childbirth, or breastfeeding. Addressing these gaps in service uptake is essential to reducing HIV incidence among both young women and newborns [10, 30].

While Nigeria has made progress in HIV prevention, key populations, particularly young women, continue to face significant challenges [31, 32]. Risky sexual behaviors, limited knowledge, and suboptimal engagement with prevention and testing services contribute to the ongoing burden of the disease. The need for targeted interventions that address these challenges is critical to achieving global HIV reduction goals [33–35].

Previous studies in Nigeria have identified general factors influencing HIV knowledge and testing, such as education, geography, and socioeconomic status. However, significant gaps remain regarding comprehensive trend analyses specifically among adolescent women. This study narrows the existing research by critically analyzing how HIV testing trends evolved from 2007 to 2016, particularly among this vulnerable demographic.

This study assessed the trends in HIV-related knowledge, behaviors, and HIV testing determinants among young women aged 15–24 in Nigeria. By examining shifts over time, the study will provide insights into the effectiveness of past interventions and identify areas requiring policy and programmatic adjustments. Understanding these evolving patterns is essential for strengthening HIV control efforts and improving access to sexual and reproductive health services for young women in Nigeria. Understanding the determinants of HIV testing among adolescent women is essential for improving public health outcomes in Nigeria. This research specifically informs targeted policy adjustments and effective educational and healthcare interventions aimed at increasing testing rates, thus potentially reducing new HIV infections.

## Methodology

**Fig. 1:**
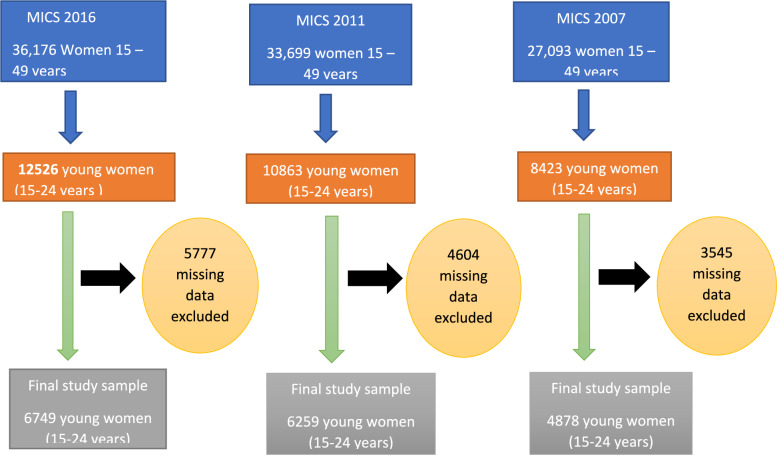
Flowchart representing the inclusion and exclusion process of young women as study participants from the MICS data 2007, 2011, and 2016 among women 15–49 years in Nigeria

The study used secondary data from the Nigeria Multiple Indicator Cluster Surveys (MICS) for 2007, 2011, and 2016. [36–38]. Data from MICS surveys (2007, 2011, 2016) were harmonized by aligning similar indicators across surveys. Adjustments were made to account for minor variations in survey questions or response options through thorough cross-validation. Each MICS survey employed a standardized two-stage stratified cluster sampling method. The first stage involved selecting enumeration areas (clusters) from census frames. In the second stage, households within these clusters were systematically sampled, ensuring national representation of the target demographic (women aged 15–24 years). There was stratification of urban and rural for the different sampled clusters. This study used data extracted from women aged 15–24 years who indicated that they had undergone HIV testing as shown in Fig. 1 below. In addition, this study applied the Health Belief Model (HBM), which posits that an individual's likelihood to engage in preventive health behaviors, such as HIV testing, depends on perceived susceptibility, perceived severity of the illness, perceived benefits of preventive action, and perceived barriers to taking action.

## Measurement of variables

### The outcome/dependent variable

The outcome variable of interest was HIV testing which is a binary variable (Tested or Not Tested).

### Independent variables

The independent variables in the study included; geopolitical zones, education status, total childbirth, marital status, status of circumcision, residential status, age at first sexual intercourse, HIV preventive behaviors and HIV-related knowledge.

HIV-related knowledge was measured by three indicators namely knowledge of HIV preventive methods, knowledge on prevention of mother-to-child transmission and comprehensive knowledge of HIV. Knowledge of HIV preventive methods was generated from respondents’ knowledge that people can prevent HIV infection by using a condom correctly every time they have sexual intercourse and by having one uninfected partner. Prevention of mother-to-child transmission was ascertained by respondents' knowledge that AIDS can be transmitted from mother to child during pregnancy, at delivery and through breast milk. Comprehensive knowledge of HIV in this study refers to knowing that a healthy-looking person can have HIV/AIDS; and knowing that one cannot get infected with HIV through supernatural means, mosquito bites and through sharing food with an HIV-infected person.

HIV preventive behavior was assessed based on multiple sexual partnerships, non-spousal sex and non-condom use at last sexual intercourse. Multiple sexual partnership was defined as having more than one sexual partner while sex with a non-marital, non-cohabiting partner was defined as non-spousal sex.

A composite score of all the component factors under HIV knowledge, behavior and testing was computed, each component factor was assigned a score of 0 for poor HIV-related knowledge, HIV preventive behavior, and 1 for good HIV-related knowledge, HIV preventive behavior. A total score for each respondent of 60% and above was determined as good HIV-related knowledge, HIV preventive behavior, and below 60% as poor HIV-related knowledge, HIV preventive behavior.

### Statistical analysis

Sample weights were applied during the analyses to adjust for disproportionate sampling to ensure representativeness and obtain less biased population parametric estimates. In descriptive statistics, observations from study samples were represented using frequencies, while proportions represented weighted estimates for the population parameters. The Chi-square test was used to assess bivariable associations. A survey-weighted multiple logistic regression analysis was performed to predict the relationship between the dependent and independent variables. Logistic regression models were built by adding each independent variable based on the statistical significance until a robust predictive model was arrived at. Multi-collinearity was assessed using the variance inflation factor and interaction of the independent variables, and independent variables that depicted multi-collinearity were excluded from the model. A P-value less than 0.05 was used to indicate the level of statistical significance. All the analysis was done using STATA 17 Corp software. Multiple logistic regression was employed due to its capacity to control for multiple confounders simultaneously, thus providing adjusted odds ratios for each predictor. Additionally, binary logistic regression was performed as a sensitivity analysis, producing consistent results and validating the robustness of our findings.

## Results

### Trends in HIV-related knowledge

Trends from 2007 to 2016 indicated persistent regional disparities and uneven progress in HIV testing rates among adolescent women, emphasizing the necessity of a deeper investigation into the determinants influencing these trends. The proportion of young women with good knowledge about HIV showed an increase across the survey years. In 2007, 37.4% of young women had good knowledge about HIV. This figure increased slightly to 39.5% in 2011 and reached 45.3% in 2016.

### Trends in HIV preventive behaviors

The proportion of young women who exhibited good behavior towards HIV prevention increased consistently over the survey years. In 2007, 74% of young women demonstrated good behavior, which rose to 78.0% by 2011 and 86% in 2016.

### Trends in HIV testing

**Fig. 2:**
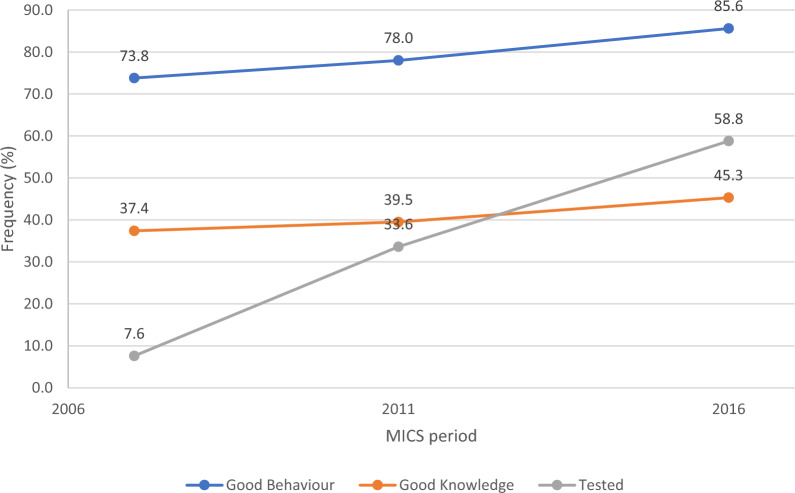
Trend of HIV testing, knowledge, and behavior among young women in Nigeria (2007 to 2016)

In Fig. 2, the proportion of young women who tested for HIV showed a steady and steeper increase across the survey years. In 2007, only 7.6% of young women had undergone HIV testing. This proportion rose steeply to 33.6% by 2011 and further increased to 58.8% in 2016.

Table 1 shows the association between the proportion of young women aged 15–24 years who tested for HIV and their background characteristics, from 2007 to 2016. At the bivariate level, the only factors found to be significantly and consistently associated with HIV testing among respondents in each of the three surveys were being from the south-east geopolitical zone, having at least a secondary level of education, urban residential status, aged between 20 and 24 years at first sexual intercourse and good knowledge of HIV. Other factors that were significantly associated with higher levels of HIV testing in some of the surveys, particularly the 2016 MICS, included young women with fewer childbirths (1–3), no history of child mortality, formerly married or never married status, non-circumcision, and high engagement in risky sexual behavior.

**Table 1:** Proportion of women who tested for HIV and selected background characteristics, MICS 2007 to 2016

Study variable	2016		2011		2007	
	p-value/percentage	n	p-value/percentage	n	p-value/percentage	n
Geopolitical zones	0.000		0.000		0.000	
Northcentral	46.0	408	24.8	207	31.9	58
Northeast	28.6	157	10.0	91	15.2	15
Northwest	15.9	210	5.7	73	8.6	14
Southeast	50.3	187	26.8	138	48.1	37
South-south	46.6	324	22.7	201	19.6	35
Southwest	35.7	152	23.6	111	29.8	28
Education status	0.000		0.000		0.000	
None	13.9	203	4.3	76	12.6	31
Primary	30.2	147	14.3	90	20.3	40
Secondary +	47.3	1088	26.3	655	33.0	116
Total childbirth	0.000		0.000		0.092	
1–3	29.3	572	13.5	305	24.3	178
4 and above	18.2	35	5.2	15	14.8	9
Total child death	0.000		0.000		0.308	
No child	35.5	1353	17.9	759	24.6	161
1–2	20.2	85	9.6	60	18.7	25
3 and above	0.0	0	7.1	2	16.7	1
Marital status	0.000		0.000		0.467	
Currently married	27.6	708	12.8	378	22.9	163
Formerly married	45.5	46	22.6	24	30.4	7
Never married	43.2	684	23.0	419	28.3	17
Status of circumcision	0.000		0.908		0.800	
Circumcised	36.0	254	22.5	194	28.0	53
Not circumcised	46.0	713	22.3	354	29.1	72
Residential status	0.000		0.000		0.000	
Urban	47.6	511	27.9	266	34.4	78
Rural	29.2	927	14.1	555	19.2	109
Age at first sexual intercourse	0.000		0.000		0.008	
5–14	23.5	144	9.7	75	26.8	30
15–19	36.7	839	20.2	521	23.7	100
20–24	54.9	191	37.6	130	44.0	22
HIV preventive behavior	0.146		0.076		0.000	
Poor behavior	35.7	369	18.4	234	28.4	29
Good behavior	33.3	1069	16.2	587	22.8	158
HIV-related knowledge	0.000		0.000		0.000	
Poor knowledge	22.0	455	13.1	378	19.0	89
Good knowledge	45.2	983	22.3	443	30.2	98

Young women from the northwest geopolitical zone were significantly associated with a lower percentage of HIV testing, ranging from 5.7% to 15.9% over the survey period 2007 to 2016 compared with 26.8% to 50.3% among those from the south-east geopolitical zone. Young women with no education were significantly associated with a lower percentage of HIV testing, ranging from 4.3% to 13.9% over the survey period 2007 to 2016 compared with 26.3% to 47.3% among those with at least secondary education. The analysis further reveals a significant association between young women from rural areas and HIV testing with a lower proportion for HIV testing ranging from 14.1% to 29.2% over the survey period 2007 to 2016 compared with 27.9% to 47.6% among their counterparts from urban areas. Furthermore, those who had poor knowledge about HIV were significantly associated with a lower percentage of HIV testing, ranging from 13.1% to 22.0% over the survey period 2007 to 2016 compared with 22.3% to 45.2% among their counterparts with good knowledge about HIV.

Young women with 4 children and above were significantly associated with lower rates of HIV testing compared to those with fewer childbirths (1–3). Young women who had not experienced any child mortality (no child death) were significantly associated with higher chances of HIV testing compared to those who had lost at least 1 child. Young women who were currently married registered lower cases of HIV testing, ranging from 12.8% to 27.6% over the survey period 2007 to 2016 compared with 22.6% to 45.5% among those who were formerly married.

The higher testing rates in the Southeast geopolitical zone may reflect relatively better access to education and healthcare infrastructure compared to regions such as the Northwest, highlighting the need for region-specific interventions.

Table 2 presents results for a multivariate logistic regression model to establish factors associated with HIV testing. Consistent factors associated with HIV testing across all three years of the survey included North West and South-South geopolitical zones, rural residential status and good knowledge about HIV. Results also show that in the two most recent surveys, from 2011 to 2016, young women with primary education were significantly associated with reduced odds of HIV testing compared to those with at least secondary education when compared with those with no formal education.

**Table 2:** Results from multivariate logistic regression of socioeconomic factors associated with HIV testing, MICS 2007 to 2016

Study variable	Year		
	2016	2011	2007
Geopolitical zones (Ref: Northcentral)			
Northeast	0.6*	0.4**	0.4
	(0.4–0.9)	(0.3–0.7)	(0.1–1.4)
Northwest	0.3***	0.4***	0.2**
	(0.2–0.5)	(0.3–0.7)	(0.1–0.6)
Southeast	0.6**	0.8	1.6
	(0.5–0.9)	(0.6–1.2)	(0.8–3.6)
South-south	0.5***	0.7*	0.4*
	(0.3–0.6)	(0.5–0.9)	(0.2–0.9)
Southwest	0.3***	0.5***	0.5
	(0.2–0.4)	(0.3–0.7)	(0.2–1.0)
Education status (Ref: None)			
Primary	1.6*	2.2**	1.0
	(1.1–2.3)	(1.3–3.7)	(0.4–2.5)
Secondary +	3.3***	5.6***	1.8
	2.4–4.6	3.6–8.8	0.8–4.2
Marital status (Ref: Currently married)			
Formerly married	1.7	1.5	1.7
	(1.0–3.0)	(0.8–2.7)	(0.4–6.1)
Never married	1.0	0.8	1.3
	(0.8–1.3)	(0.9–1.4)	(0.8–2.0)
Residential status (Ref: Urban)			
Rural	0.7***	0.5***	0.4***
	(0.5–0.8)	(0.4–0.7)	(0.2–0.6)
HIV preventive behavior (Ref: Poor behavior)			
Good behavior	1.8***	1.7***	1.5
	(1.4–2.2)	(1.4–2.3)	(0.6–3.4)
HIV-related knowledge (Ref: Poor knowledge)			
Good knowledge	2.1***	1.3**	2.0**
	(1.8–2.6)	(1.1–1.6)	(1.3–3.2)

^***^p < 0.001, **p < 0.01, *p < 0.05

Young women with good HIV preventive behavior were significantly associated with higher odds of HIV testing, ranging from 1.7 times higher in 2011 to 1.8 times higher in 2016 compared with young women with poor HIV preventive behavior.

Multiple logistic regression allowed simultaneous control of multiple predictors influencing HIV testing. Binary logistic regression analyses confirmed consistency of results, strengthening the methodological validity.

## Discussion

This study assessed the trends in HIV-related knowledge, behaviors, and HIV testing determinants among young women aged 15–24 in Nigeria. The results align with similar studies in Nigeria and other sub-Saharan African countries, highlighting education, geographical location, and behavioral factors as key determinants of HIV testing. Notably, [34, 39] demonstrated similar associations between education and HIV testing among adolescent girls, validating our findings. This study demonstrated a notable and consistent decline in risky sexual behavior among young women from 2007 to 2016, with the proportion of women engaging in poor HIV preventive behaviors nearly halving by 2016. This trend is praiseworthy and likely reflects the concerted efforts of governmental bodies and collaborative partners in combating the HIV/AIDS pandemic. By reducing the incidence of risky sexual behavior among young women, there is a ripple effect on various aspects of public health. This includes a potential decrease in new HIV infections, lower demands on healthcare systems for HIV/AIDS-related care, and possibly a shift in focus towards preventive measures and comprehensive sexual education programs [34]. Also, the decline in risky sexual behavior extends beyond mere statistical improvements. It suggests a potential decrease in HIV transmission rates, thereby alleviating the burden associated with the care and management of individuals living with HIV/AIDS. This reduction in risky behavior not only impacts individual health outcomes, but also has broader implications for public health interventions and resource allocation.

Moreover, the findings of this study underscore the importance of sustained efforts in promoting safe sexual practices among young women. Continued investment in education, access to healthcare services, and community-based interventions can further reinforce this positive trend and contribute to the overall well-being of the population. Comparatively, the proportion of adolescent girls and young women engaging in risky sexual behaviors in this study consistently remained lower than that reported in similar studies conducted in Nigeria and Ghana [35, 39]. This disparity suggests potential variations in factors influencing sexual behavior among adolescent population across different regions and highlights the need for targeted, context-specific interventions to address these differences effectively.

The study also revealed a significant decline in the proportion of young women with poor knowledge of HIV, from 2007 to 2016, indicating a steady improvement in HIV knowledge among this population [40]. This enhancement in knowledge likely contributed to the decline in risky sexual behaviors observed among the same population, as individuals with a better understanding of HIV are more likely to engage in protective behaviors. This correlation between HIV-related knowledge and risky sexual behavior is supported by a study in Ibadan, Nigeria, which found that improved HIV knowledge was associated with a decrease in risky sexual behavior [29]. The implications of these findings are substantial, highlighting the importance of comprehensive HIV education and awareness programs targeting adolescents and young women. By investing in knowledge acquisition and awareness creation, we can empower young people to make informed decisions about their sexual health, ultimately reducing the risk of HIV transmission and promoting healthier behaviors. These findings underscore the need for sustained efforts to improve HIV knowledge and reduce risky sexual behavior among young women, a critical step towards achieving improved public health outcomes.

The proportion of young women who have tested for HIV showed a remarkable increase from 2007 to 2016, which may be attributed to the concurrent increase in HIV knowledge during the same period [39]. This upward trend is likely a result of the concerted efforts by governments and development partners to promote HIV awareness and testing. While the 2016 figure is comparable to that reported in Eswatini and Ethiopia, it surpasses the findings from The Gambia, highlighting potential methodological differences [40, 41]. Nevertheless, the figures indicate that a substantial proportion of young women remain untested, falling short of the ambitious 90–90-90 targets for HIV care as at 2020. This disparity underscores the need for intensified efforts to expand HIV testing services, particularly among adolescents, to ensure early detection, treatment, and ultimately, control of the pandemic. To achieve this, innovative strategies such as school-based testing, community outreach, and technology-enabled testing platforms must be explored and scaled up to reach the remaining gap in HIV testing among adolescents and young women.

This study revealed that geopolitical zones, education status, residential status, and knowledge of HIV are significant predictors of HIV testing among adolescent girls and young women. Notably, adolescents and young women from the North East, North West, South East, South-South, and South West geopolitical zones were less likely to undergo HIV testing compared to their counterparts from the North Central zone. A possible reason for this disparity may be the varying levels of access to healthcare services and HIV testing facilities across different geopolitical zones.

Young women with primary education were significantly more likely to undergo HIV testing compared to those with no formal education, and this likelihood increased substantially among those with secondary education. This finding suggests that higher educational status is a strong predictor of HIV testing among adolescents and young women, consistent with studies in Ethiopia and The Gambia [41, 42]. This has important public health implications, as it highlights the need to prioritize education as a critical component of HIV prevention and treatment strategies. By promoting education, we can empower young women to make informed decisions about their health, increase their uptake of HIV testing services, and ultimately reduce the spread of HIV. Furthermore, these findings underscore the importance of integrating HIV education and testing into school-based programs, ensuring that adolescents and young women have access to accurate information and services to protect their sexual health. Throughout the study period, young women residing in rural areas consistently showed a lower likelihood of undergoing HIV testing compared to their urban counterparts. This is corroborated by findings in studies from the US and China [43, 44]. This disparity may be attributed to the limited availability and accessibility of healthcare services, including HIV testing facilities, in rural areas. This finding has significant public health implications, as it highlights the need to address the disparities in healthcare access and HIV testing services between rural and urban areas. To reduce the spread of HIV and improve overall public health, it is essential to increase access to HIV testing and treatment services in rural areas, potentially through mobile health clinics, community-based testing programs, and other innovative strategies. By bridging this gap, we can ensure that all adolescents, regardless of their geographical location, have equal opportunities to know their HIV status and receive appropriate care.

Young women who exhibited good HIV preventive behavior and had good knowledge of HIV were more likely to undergo HIV testing compared to their counterparts with poor behavior and poor knowledge, respectively. This suggests that adolescents who engage in responsible behaviors and have a better understanding of HIV are more likely to take proactive steps towards knowing their HIV status. These findings highlight the importance of integrating behavioral interventions and HIV education into adolescent health programs, to not only improve HIV knowledge but also promote healthy behaviors and increase uptake of HIV testing services. By addressing these factors, we can empower adolescents to make informed decisions about their health and reduce the risk of HIV transmission.

## Conclusion

In conclusion, this study demonstrates a promising trend of declining risky sexual behavior and improving HIV knowledge among young women in Nigeria. The findings highlight the importance of sustained efforts in promoting safe sexual practices, comprehensive HIV education, and access to HIV testing services. The study's results underscore the need for targeted interventions to address regional and educational disparities in HIV testing and knowledge. The study observed significant increases in HIV testing rates among adolescent women from 7.6% in 2007 to 58.8% in 2016. Factors such as higher education, urban residence, and improved HIV knowledge significantly increased testing uptake, underlining the importance of targeted educational and healthcare strategies.

## Limitations of the study

Limitations of this study include the reliance on self-reported data which may introduce recall bias. The exclusion of the latest MICS 2021 data is also a limitation. Future studies should include the latest available data and qualitative analyses to further explore sociocultural dynamics influencing HIV testing. The research relied on self-reported data obtained from the Nigeria Multiple Indicator Cluster Surveys (MICS) where participants might have experienced poor recall capabilities and a tendency for desirable responses. Participants may distort their reports regarding HIV-related knowledge and behaviors as well as their testing experiences thus creating errors in study outcomes.

The analysis employed MICS records from 2007, 2011, and 2016 which might not adequately represent the latest HIV prevention progress and test implementation developments throughout Nigeria. Recent HIV-related data about young women are missing so researchers cannot evaluate current changes in their knowledge and behaviors.

The researchers only analyzed the relationships between HIV-related knowledge, behavior and testing through a designated investigation point in this study. The study cannot demonstrate cause–effect relationships between variables thus obscuring understanding of which factors directly affect societal changes.

## Recommendations

- Sustain and intensify efforts to promote safe sexual practices and comprehensive HIV education among young women.
- Implement targeted interventions to address regional and educational disparities in HIV testing and knowledge.
- Prioritize education as a critical component of HIV prevention and treatment strategies.
- Integrate HIV education and testing into school-based programs.
- Increase access to healthcare services, including HIV testing facilities, in rural areas.
- Explore innovative strategies, such as mobile health clinics and community-based testing programs, to reach adolescents in rural areas.
- Continue to monitor and evaluate the effectiveness of HIV prevention and treatment efforts among young women in Nigeria.

By implementing these recommendations, we can improve the health and well-being of young women in Nigeria and contribute to the global effort to control the HIV/AIDS pandemic. (1) Systems: strengthen healthcare infrastructure, particularly in rural and underserved regions, to facilitate easier access to HIV testing and counseling services; (2) Programmes: implement comprehensive school-based HIV testing and educational programs to ensure adolescents are reached effectively and early; and (3) Processes: develop culturally sensitive community engagement initiatives to reduce stigma and enhance community acceptance of HIV prevention and testing services.

## Data Availability

The data that support the findings of this study are available on request from the corresponding author. The data are not publicly available due to privacy or ethical restrictions. But can be accessed on request from https://mics.unicef.org/.

## Abbreviations

HIV: Human Immune Deficiency Virus (HIV)
VCT: Voluntary counselling and testing
PMTCT: Prevention of mother-to-child transmission
MICS: Multiple indicator cluster surveys
AIDS: Acquired immune deficiency syndrome
UNICEF: United Nations Children’s Fund
